# Comparative functional analysis of proteins containing low-complexity predicted amyloid regions

**DOI:** 10.7717/peerj.5823

**Published:** 2018-10-30

**Authors:** Bandana Kumari, Ravindra Kumar, Vipin Chauhan, Manish Kumar

**Affiliations:** 1 Department of Biophysics, University of Delhi South Campus, New Delhi, India; 2 Department of Genetics, University of Delhi South Campus, New Delhi, India; 3 Current affiliation: Centre for Neuroscience, Indian Institute of Science, Bangalore, India

**Keywords:** Low complexity regions, Amyloids, Amino acid runs, Functional annotation

## Abstract

**Background:**

In both prokaryotic and eukaryotic proteins, repeated occurrence of a single or a group of few amino acids are found. These regions are termed as low complexity regions (LCRs). It has been observed that amino acid bias in LCR is directly linked to their uncontrolled expansion and amyloid formation. But a comparative analysis of the behavior of LCR based on their constituent amino acids and their association with amyloidogenic propensity is not available.

**Methods:**

Firstly we grouped all LCRs on the basis of their composition: homo-polymers, positively charged amino acids, negatively charged amino acids, polar amino acids and hydrophobic amino acids. We analyzed the compositional pattern of LCRs in each group and their propensity to form amyloids. The functional characteristics of proteins containing different groups of LCRs were explored using DAVID. In addition, we also analyzed the classes, pathways and functions of human proteins that form amyloids in LCRs.

**Results:**

Among homopolymeric LCRs, the most common was Gln repeats. LCRs composed of repeats of Met and aromatic amino acids were amongst the least occurring. The results revealed that LCRs composed of negatively charged and polar amino acids were more common in comparison to LCRs formed by positively charged and hydrophobic amino acids. We also noted that generally proteins with LCRs were involved in transcription but those with Gly repeats were associated to translational activities. Our analysis suggests that proteins in which LCR is composed of hydrophobic residues are more prone toward amyloid formation. We also found that the human proteins with amyloid forming LCRs were generally involved in binding and catalytic activity.

**Discussion:**

The presented analysis summarizes the most common and least occurring LCRs in proteins. Our results show that though repeats of Gln are the most abundant but Asn repeats make longest stretch of low complexity. The results showed that potential of LCRs to form amyloids varies with their amino acid composition.

## Introduction

Low complexity regions (LCRs) in proteins are either composed of repeats of single amino acids or short amino acid motifs (Wootton & Federhen, 1996). Because of enrichment of one or a few amino acids, LCRs are characterized by its low information content. Statistical analysis suggests that up to 25% of proteome are found within LCRs (Wootton, 1994) and their abundance is more than what expected as random (Alba, Tompa & Veitia, 2007). The well-characterized examples of LCRs are single amino acid repeats, also called as runs (Harbi, Kumar & Harrison, 2011). Proteins carrying Ala, Lys and Pro repeats are known to play key role in several important biological processes (BP) such as development, immunity, reproduction and cellular localization (Albrecht et al., 2004; Toro Acevedo et al., 2017). The compositional bias of LCRs makes them prone to undergo expansion or contraction, which ultimately influences the function of protein in which it is present. For example, in many species length variation of LCRs affects circadian rhythm duration (Avivi et al., 2001) and phenotypic characters (Michael et al., 2007). They are also of medical interest, because uncontrolled expansions of such regions may induce self-aggregation and formation of amyloid fibrils in eukaryotes (Michelitsch & Weissman, 2000). Amyloids are fibrous protein forms that are assembled of cross β-sheet structure (Chiti & Dobson, 2006) and show high degree of protease resistance. Gain-of-toxic functions by amyloid fibrils are known to cause several devastating human diseases which include, but are not limited to, Type II diabetes, rheumatoid arthritis, and several progressive neurodegenerative disorders such as Alzheimer’s disease, Parkinson’s disease, Spinocerebellar ataxias and Huntington’s disease (Michelitsch & Weissman, 2000; Kreil & Ouzounis, 2003; Gunawardena & Goldstein, 2005). Many studies demonstrated that amyloids are commonly formed in Gln and/or Asn rich domains (Scherzinger et al., 1997; Warrick et al., 1998; Chow, Paulson & Bottomley, 2004). These regions form mutation-linked pathological amyloids as well as the functional amyloids, induced by a specific stimulus. For example, in TDP-43 (TAR DNA-binding protein) single residue substitution mutation in the Gln/Asn-enriched LCR forms irreversible protein aggregates which are involved in amyotrophic lateral sclerosis and frontotemporal lobar dementia (Neumann et al., 2006; Johnson et al., 2009). In Huntington patients, expansion of poly-Gln runs in huntingtin protein is responsible for formation of intranuclear and cytoplasmic aggregates, which result in Huntington disease (DiFiglia et al., 1997; Huang et al., 1998). In yeast, LCRs of Cdc19, an isoform of pyruvate kinase, and termination proteins, Nab3 and Nrd1 assemble to form functional amyloid (O’Rourke et al., 2015; Grignaschi et al., 2018). Moreover, yeast prions [*PUB1*] and [*SUP35*] are known functional amyloids which form microtubule-associated complex important for translation (Li et al., 2014; Nizhnikov et al., 2016). The peptide GNNQQNY results in aggregation of SUP35 (Garbuzynskiy, Lobanov & Galzitskaya, 2010).

Despite the importance in several pathological conditions, the functional properties of proteins containing LCRs, and impact of constituent amino acids in the propensity of LCR toward amyloid formation is not worked out in much detail. Also, LCR have been generally excluded from wet lab structure-function correlation experiments and in silico functional analysis due to their less amenability of crystallization and difficulty in sequence alignment, respectively. This is also a major reason toward availability of less information on impact of LCRs on protein functions.

In this paper, we classified LCRs according to their amino acid composition. LCRs which were composed of more than one amino acid but having same physico-chemical properties were grouped into four classes: positively charged, negatively charged, polar and hydrophobic. We did comparative functional analysis of proteins containing LCRs consisting of a single amino acid and amino acid of similar physico-chemical properties. Additionally, for all LCR groups, we predicted the propensity of amyloid formation and analyzed their compositional patterns and optimum size. We also predicted amyloid formation in LCRs of human proteome and annotated the pathways, classes and functions in which they participate. Overall, results of this study will further increase our understanding on LCRs in triggering functional variations and formation of amyloids.

## Materials and Methods

### Dataset compilation

For this study, we downloaded 553,231 protein sequences from SwissProt. To remove redundancy among proteins in the dataset we used CD-HIT (Li & Godzik, 2006) with the aim that no two proteins have ≥40% pairwise sequence identity between them. Any proteins containing non-standard amino acid residues were also removed. Finally we obtained 85,381 protein sequences in total.

### Extraction and classification of LCRs

We next identified LCRs in the protein sequences using SEG which uses Shannon’s entropy to search region of low complexity in a protein sequence. During the LCR identification process, SEG collects all possible subsequences of length *L* having the local sequence complexity ≤K1. All overlapping subsequences having sequence complexity ≤K1 are merged in both directions till the complexity of contig created by overlapping subsequences lie below ≤K2 (Kumari, Kumar & Kumar, 2015). In this work we used default values of SEG (*L* = 12, *K*1 = 2.2, *K*2 = 2.5). Among all LCRs we kept only those which has at least three amino acids. The final dataset had 186,637 LCRs obtained from 59,821 non-redundant proteins. The complete LCR dataset was divided into two sets: Set-I which contained LCRs composed of runs of single amino acid (Harbi, Kumar & Harrison, 2011) and Set-II contained LCRs which were composed of more than one type of amino acids but of similar physico-chemical property. Thus, Set-I has 20 different subsets of LCR, each corresponds to a distinct amino acid. Depending on the functional groups of amino acids, Set-II was also further divided into positive charged (Arg, Lys), negative charged (Glu, Asp), polar (Arg, Lys, Asn, Gln, Asp, Glu) and hydrophobic (Cys, Ile, Leu, Met, Phe, Trp, Val) LCRs.

### Functional enrichment

In order to gain insights into the functions, we performed GO-term enrichment analyses on all proteins containing LCRs. This analysis was done using DAVID (Huang Da, Sherman & Lempicki, 2009), which can handle a number of heterogeneous annotation terms (e.g., GO terms, protein domains, pathways and so on) or gene classes and thus helps in visualization of the larger biological picture. For functional analysis we used complete set of SwissProt proteins as background.

### Extraction of amyloids in LCRs

#### Data I

In protein sequences containing LCRs, Waltz (Maurer-Stroh et al., 2010) was used to find the potential amyloid forming regions with default parameters. Waltz can efficiently recognize local amyloid propensity and differentiate them from “amorphous” aggregates,“proto fibrils,” or the mixture of all. In order to find LCRs, which may form aggregates, LCRs were mapped with Waltz prediction and regions common in both were considered as amyloidogenic LCR. Using this approach, amyloidogenic regions were retrieved from LCR sets. To achieve high reliability, only amyloid regions with at least three amino acids were considered for analysis.

#### Data II

We also collected experimentally annotated amyloid proteins from AmyPro database (Varadi et al., 2018). AmyPro had information about 174 amyloid regions distributed in 126 protein sequences. We located LCRs in these 126 proteins using SEG and found that 76 protein stretches had common LCRs and amyloid regions (hereafter named as *Data II*). In AmyPro 70 proteins were belonged to human. Out of 70, in 31 proteins we found overlapping LCR and amyloid regions. This dataset is named as *Data IIh* in this manuscript and was used for functional analysis of human proteins that forms amyloids.

### Prediction of amyloids in LCRs of human proteome

In order to study the aggregation tendency of LCRs in human proteome, we used human proteome compilation of HPRD (Keshava Prasad et al., 2009). Using default parameters of SEG, we found LCRs in 23,727 proteins out of total 30,046 proteins. Subsequently amyloid regions were predicted in LCR-containing proteins with Waltz.

### Functional annotation of human proteins with amyloids in LCRs

Using Protein Analysis THrough Evolutionary Relationships (PANTHER) (Mi, Muruganujan & Thomas, 2013), we analyzed the classes, pathways and functions of human protein which were predicted to have amyloidogenic LCRs. PANTHER does gene annotations on the basis of evolutionary relationships, which were taken from Gene Ontology Reference Genome project.

## Results

### Compositional trends of LCRs: in general and in amyloids

We first checked prevalent amino acids in LCRs and analyzed whether amino acid composition of LCR affects their aggregation tendency. We did compositional analysis for each LCR sets categorized on the basis of homopolymeric runs, charge and hydrophobicity. The most common homopolymeric runs were *poly*Gln, *poly*Asn, *poly*Ser, *poly*Ala, *poly*Glu and *poly*Pro (in decreasing order) (Table 1). *poly*Leu, *poly*Lys, *poly*Asp, *poly*Gly and *poly*Thr were also found in moderate number. The least preferable homo-repeats were *poly*Val (13 in number), *poly*Phe and *poly*Ile (11 in number), *poly*Tyr (seven in number), *poly*Cys (two in number) and *poly*Met (one in number). We observed total absence of *poly*Trp LCRs in our dataset (Table 1). The compositional trend analysis on Set-II LCRs revealed that the number of positively charged LCRs was *ca.* 1/3rd of the negative charged LCRs. The results also showed the number of LCRs composed of polar amino acids was more than hydrophobic amino acids (Table 1).

**Table 1:** Distribution of low complexity regions and amyloids predicted in them.

		Ala	Cys	Ile	Leu	Met	Val	Trp	Phe	Arg	Lys	Asn	Asp	Glu	Gln	Gly	His	Pro	Ser	Thr	Tyr
**Total LCRs**	LCRs	273	2	11	142	1	13	0	11	48	102	352	108	227	391	181	49	205	330	128	7
	Proteins	265	2	11	141	1	13	0	10	47	100	315	108	225	351	177	49	195	325	124	7
	Residue	2005	12	55	849	6	61	0	78	272	627	5967	851	1721	4789	1348	364	1481	2435	996	35
**Amyloids in LCRs**	Proteins	30 (123)	1 (1)	11 (11)	42 (88)	0 (0)	5 (7)	0 (0)	10 (10)	0 (13)	1 (41)	54 (253)	1 (47)	4 (80)	39 (244)	3 (87)	1 (20)	0 (93)	15 (152)	6 (71)	7 (7)
	Residue	76 (1092)	1 (1)	55 (202)	171 (1580)	0 (0)	18 (60)	0 (0)	78 (121)	0 (118)	1 (1415)	170 (11051)	1 (695)	4 (1098)	90 (6891)	3 (1392)	1 (194)	0 (1261)	28 (2716)	9 (1983)	35 (111)
										**Positively charged**			**Negatively charged**								
**Total LCRs**	LCRs									211			629								
	Proteins									203			614								
	Residue									1360			5534								
**Amyloids in LCRs**	Proteins									1 (74)			8 (224)								
	Residue									1 (2140)			9 (3011)								
			**Hydrophobic**							**Polar**											
**Total LCRs**	LCRs		709							2756											
	Proteins		698							2390											
	Residue		6164							33000											
**Amyloids in LCRs**	Proteins		506 (590)							174 (1189)											
	Residue		4302 (10239)							437 (28311)											

**Note:**

Values in paranthesis are total number of amyloid forming protein/residue under LCRs in total dataset. The categorization of amino acids on basis of physico-chemical properties are as follows: Positively charged: Arg and/or Lys; Negatively charged: Glu and/or Asp; Hydrophobic: any of Cys, Ile, Leu, Met, Phe, Trp, Val or their combination; Polar: any of Arg, Lys, Asn, Gln, Asp, Glu.

Prediction of amyloids in LCRs suggests that *poly*Ala, *poly*Phe, *poly*Leu, *poly*Asn, *poly*Gln, polar and hydrophobic amino acids have amyloidogenic capability. Majority of the amyloidogenic LCRs were composed of *poly*Leu, *poly*Asn and hydrophobic residues. In contrast, *poly*Cys, *poly*Asp, *poly*Glu, *poly*Lys, *poly*Gly, *poly*His, *poly*Thr and charged LCRs accounted for a very small fraction of amyloidogenic LCRs with less than 10 residues. The runs of *poly*Met, *poly*Trp, *poly*Pro and *poly*Arg were predicted to be completely lacked of amyloidogenic capability (Table 1).

**Figure 1:**
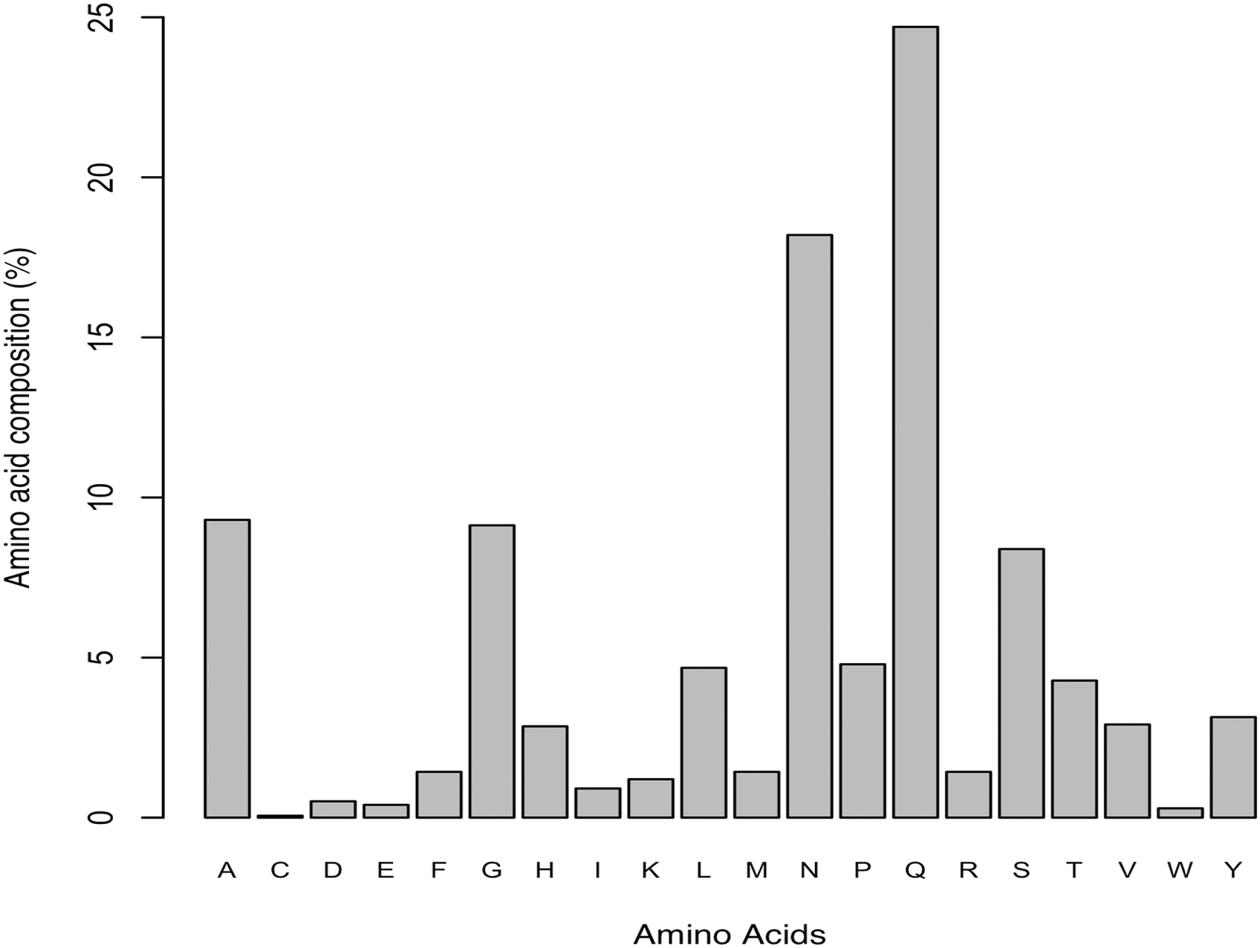
Amino acid composition of experimentally characterized amyloids found in low complexity regions (Data source: AmyPro).

In order to verify our results obtained by prediction of amyloidogenic LCRs, we repeated the analysis on effect of amino acid composition on amyloidogenesis in *Data II*, which had only experimentally verified amyloidogenic LCRs. The results revealed Gln and Asn as the most abundant; Ala, Gly and Ser as moderate; and Cys and Trp as the least preferred (Fig. 1). This observation was inline with our earlier observation on predicted amyloids in LCRs. We also identified two polar LCRs and one hydrophobic LCR in *Data II*.

### Length analysis of amyloids

**Figure 2:**
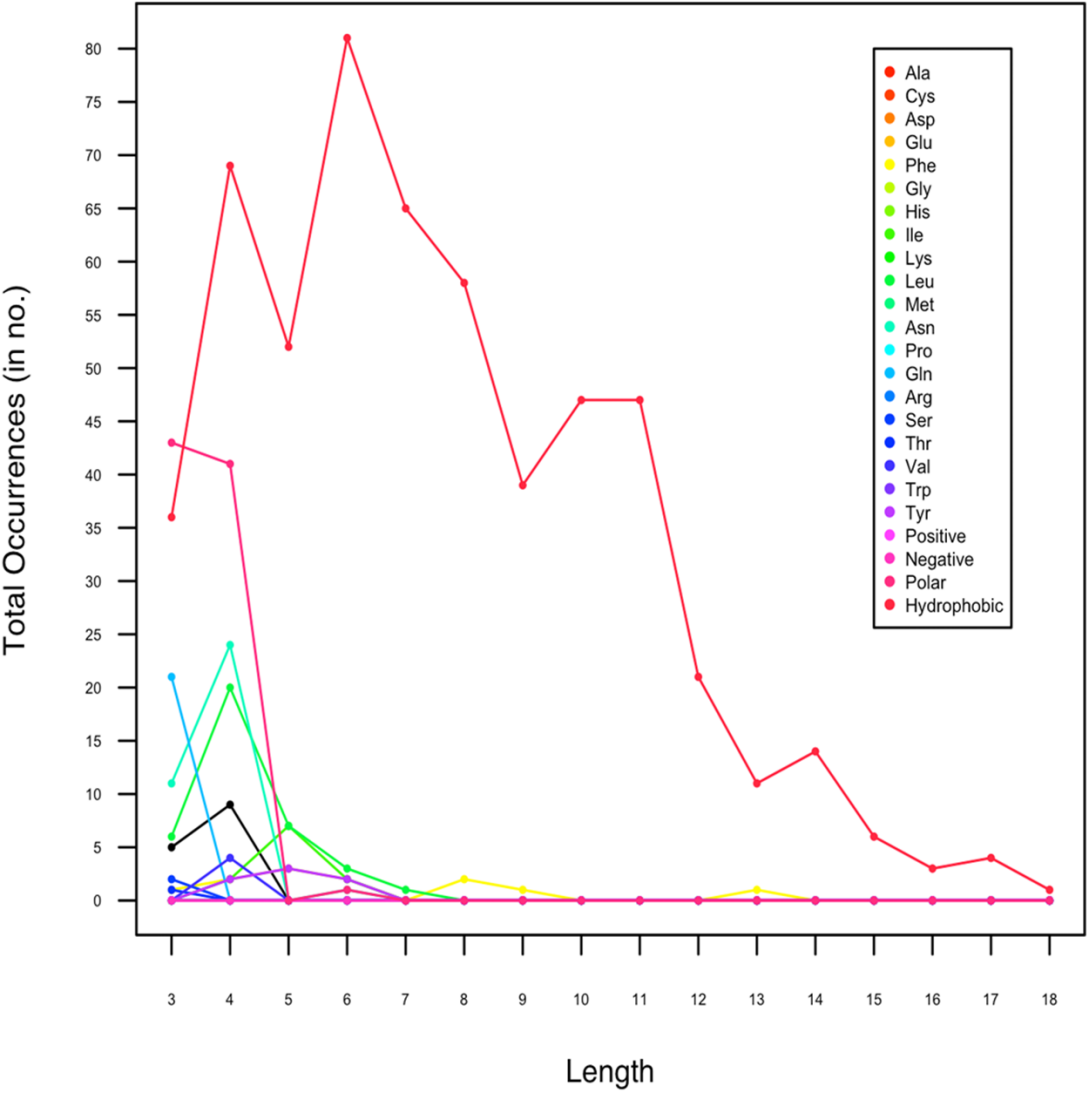
Length-wise distribution of low complexity regions in protein sequences which are predicted to form amyloids by Waltz.

In an attempt to investigate whether the amyloids have size variation, we also analyzed the length of amyloidogenic LCRs. In *Data I*, the homopolymeric runs, predicted to form amyloids, ranged between 3 and 6 AA (Fig. 2). The maximum length of amyloid forming *poly*Leu runs was 7 AA and *poly*Asn was 4 AA. For hydrophobic LCRs, shorter amyloids of length 3–11 predominated whereas the length of amyloidogenic polar LCRs were 3–4. We found that hydrophobic LCRs had the longest stretch (18 AA). As the longer hydrophobic stretches are known to be toxic (Dorsman et al., 2002; Oma et al., 2004), this indicates the toxic nature of the amyloidogenic LCRs.

In *Data II*, which contained only experimentally proven amyloidogenic LCRs, the length of LCRs composed of polar amino acid ranged between 8 and 9 and for hydrophobic LCR, it was 16. This was inline with our observation with *Data I*.

### Functional enrichment of LCRs

To study the functions in which proteins having amyloidogenic LCRs are involved, we analyzed their BP, molecular functions (MF) and cellular components (CC) abundance in each category of LCR using DAVID (https://david.ncifcrf.gov/). For our analysis we considered only the top five enriched GO-terms.

**Figure 3:**
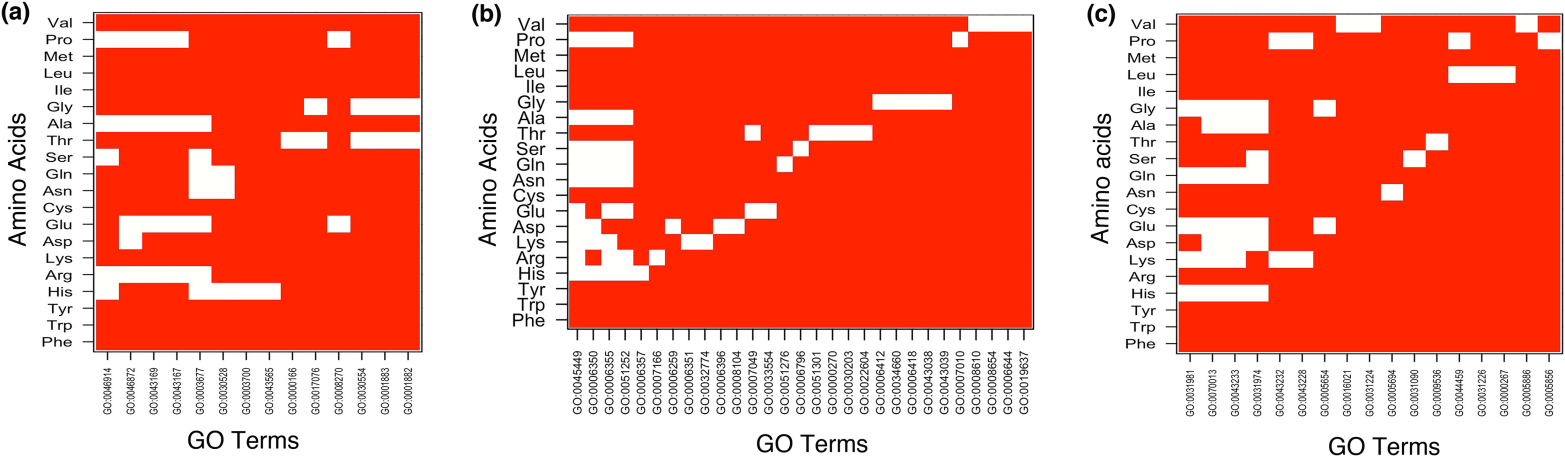
Top 5 enriched (A) molecular functions (B) biological processes and (C) cellular components in homopolymeric repeats. In Figure white color indicates the presence of GO terms while the red color indicates their absence. The description of GO terms is as follows: For Molecular Functions—GO:0000166, Nucleotide binding; GO:0001882, Nucleoside binding; GO:0001883, Purine nucleoside binding; GO:0003677, DNA binding; GO:0003700, Transcription factor activity; GO:0008270, Zinc ion binding; GO:0017076, Purine nucleotide binding; GO:0030528, Transcription regulator activity; GO:0030554, Adenyl nucleotide binding; GO:0043167, Ion binding; GO:0043169, Cation binding; GO:0043565, Sequence-specific DNA binding; GO:0046872, Metal ion binding; GO:0046914, Transition metal ion binding. For Biological Processes—GO:0000270, Peptidoglycan metabolic process; GO:0006259, DNA metabolic process; GO:0006350, Transcription; GO:0006351, Transcription, DNA-dependent; GO:0006355, Regulation of Transcription, DNA-dependent; GO:0006357, Regulation of Transcription from RNA polymerase II promoter; GO:0006396, RNA processing; GO:0006412, Translation; GO:0006418, tRNA aminoacylation for protein translation; GO:0006644, Phospholipid metabolic process; GO:0006796, Phosphate metabolic process; GO:0007010, Cytoskeleton organization; GO:0007049, Cell cycle; GO:0007166, Cell surface receptor linked signal transduction; GO:0008104, Protein localization; GO:0008610, Lipid biosynthetic process; GO:0008654, Phospholipid biosynthetic process; GO:0019637, Organophosphate metabolic process; GO:0022604, Regulation of cell morphogenesis; GO:0030203, Glycosaminoglycan metabolic process; GO:0032774, RNA biosynthetic process; GO:0033554, Cellular response to stress; GO:0034660, ncRNA metabolic process; GO:0043038, Amino acid activation; GO:0043039, tRNA aminoacylation; GO:0045449, Regulation of Transcription; GO:0051252, Regulation of RNA metabolic process; GO:0051276, Chromosome organization; GO:0051301, Cell division. For Cellular Components—GO:0000267, Cell fraction; GO:0005654, Nucleoplasm; GO:0005694, Chromosomal part; GO:0005856, Cytoskeleton; GO:0005886, Plasma membrane; GO:0009536, Plastid; GO:0016021, Integral to membrane; GO:0031090, Organelle membrane; GO:0031224, Intrinsic to membrane; GO:0031226, Intrinsic to plasma membrane; GO:0031974, Membrane-enclosed lumen; GO:0031981, Nuclear lumen; GO:0043228, Non-membrane-bounded organelle; GO:0043232, Intracellular non-membrane-bounded organelle; GO:0043233, Organelle lumen; GO:0044459, Plasma membrane part; GO:0070013, Intracellular organelle lumen.

**Figure 4:**
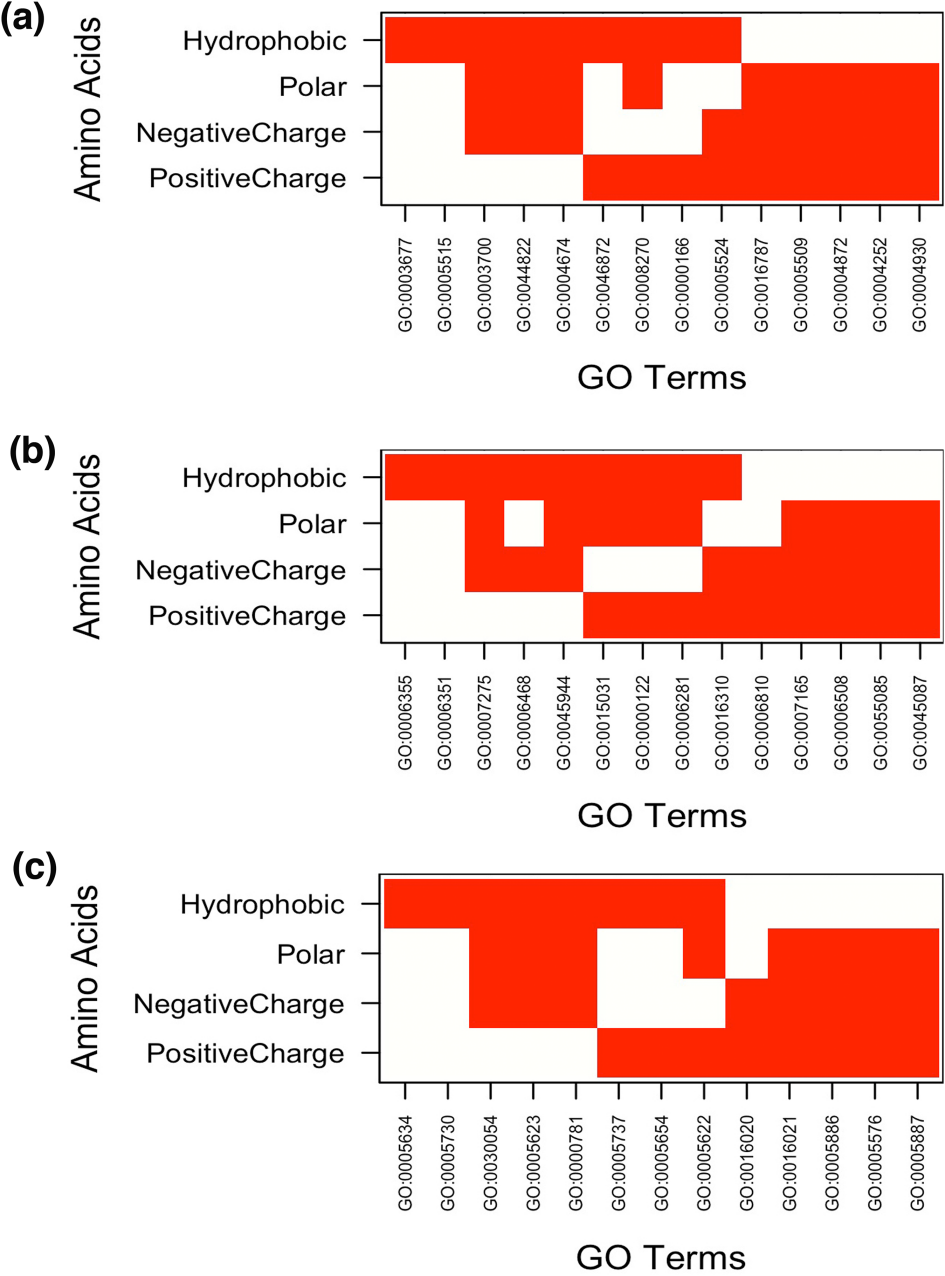
Top 5 enriched (A) molecular functions (B) biological processes and (C) cellular components in LCRs containing amino acids of similar physico-chemical properties. In Figure white color indicates the presence of GO terms while the red color indicates their absence. The description of GO terms is as follows: For Molecular Functions—GO:0000166, Nucleotide binding; GO:0003677, DNA binding; GO:0003700, Transcription factor activity, sequence-specific DNA binding; GO:0004252, Serine-type endopeptidase activity; GO:0004674, Protein serine/threonine kinase activity; GO:0004872, Receptor activity; GO:0004930, G-protein coupled receptor activity; GO:0005509, Calcium ion binding; GO:0005515, Protein binding; GO:0005524, ATP binding; GO:0008270, Zinc ion binding; GO:0016787, Hydrolase activity; GO:0044822, poly(A) RNA binding; GO:0046872, Metal ion binding. Biological Processes—GO:0000122, Negative regulation of transcription from RNA polymerase II promoter; GO:0006281, DNA repair; GO:0006351, Transcription, DNA-templated; GO:0006355, Regulation of transcription, DNA-templated; GO:0006468, Protein phosphorylation; GO:0006508, Proteolysis; GO:0006810, Transport; GO:0007165, Signal transduction; GO:0007275, Multicellular organism development; GO:0015031, Protein transport; GO:0016310, Phosphorylation; GO:0045087, Innate immune response; GO:0045944, Positive regulation of transcription from RNA polymerase II promoter; GO:0055085, Transmembrane transport. Cellular Components—GO:0005576, Extracellular region; GO:0005622, Intracellular; GO:0005623, Cell; GO:0005634, Nucleus; GO:0005654, Nucleoplasm; GO:0005694, Chromosome; GO:0005730, Nucleolus; GO:0005737, Cytoplasm; GO:0005886, Plasma membrane; GO:0005887, Integral component of plasma membrane; GO:0016020, Membrane; GO:0016021, Integral component of membrane; GO:0030054, Cell junction.

The result showed MF enrichment only in proteins having runs of His, Arg, Asp, Glu, Asn, Gln, Ser, Thr, Ala, Gly and Pro. We found that these protein subsets are strongly associated with metal ion binding, transition metal ion binding and nucleotide binding (Fig. 3A and Table S1). Both *poly*Asn and *poly*Gln were involved in similar functions, that is, DNA binding and transcription regulator activity. We observed that runs belonging to charged amino acids were involved in ion and DNA binding but LCRs composed of combination of positive and negative charged amino acids were showing additional functions, that is, protein binding (Fig. 4A and Table S1). Polar LCRs were involved in “DNA binding,” “nucleotide binding,” “ATP binding,” “protein binding” and “metal ion binding.” Perfect repeats of hydrophobic amino acids were not enriched in MF whereas LCRs composed of combination of hydrophobic amino acids were involved in “calcium ion binding,” “hydrolase activity,” “receptor activity,” “serine-type endopeptidase activity” and “G-protein coupled receptor activity” (Table S1).

Under BP category, “regulation of transcription” was the most common GO-term. Interestingly, whereas runs of all amino acids were involved in transcription, the topmost enriched function of proteins with *poly*Gly was translational activity (Fig. 3B and Table S2). We also observed that runs of different amino acids were enriched in unique BP. For example, *poly*Arg were involved in “cell surface receptor linked signal transduction,” *poly*Lys in “RNA biosynthetic process,” *poly*Asp in “protein localization,” *poly*Glu in “cellular response to stress,” *poly*Gln in “chromosome organization,” *poly*Ser in “phosphate metabolic process,” *poly*Thr in “cell division,” “peptidoglycan metabolic process,” “glycosaminoglycan metabolic process” and “regulation of cell morphogenesis,” *poly*Pro in “cytoskeleton organization” and *poly*Val in “lipid biosynthetic process” (Fig. 3B and Table S2). “Regulation of transcription, DNA-dependent” was common process in the proteins having runs of positively charged amino acids, *poly*Arg and *poly*Lys. Except GO terms “regulation of transcription, DNA-templated” and “transcription, DNA-templated,” BP of LCRs containing combinations of positively charged and combinations of negatively charged amino acids were completely different, for example, positively charged LCRs were involved in “positive regulation of transcription from RNA polymerase II promoter” whereas negatively charged LCRs were involved in “negative regulation of transcription from RNA polymerase II promoter.” Polar LCRs showed enrichment of “transcription, DNA-templated,” “phosphorylation” and “transport” but hydrophobic LCRs were enriched for “signal transduction,” “proteolysis,” “innate immune response” and “transport” (Fig. 4B and Table S2).

In case of CC, the enriched locations were “nuclear lumen,” “organelle lumen” and “membrane enclosed lumen” (Figs. 3C and 4C; Table S3). However, *poly*Leu, *poly*Pro and *poly*Val completely lacked any lumen; their enrichment terms were related to “plasma membrane.”

### Functional annotation of amyloidogenic LCRs in human proteins

We also analyzed the broad spectrum of functions for each human protein containing predicted amyloidogenic LCRs in terms of GO slim functional categories *viz.*, BP, MF, CC and functional classes using PANTHER. Under BP category, we noticed the annotation terms “biological regulation,” and “response to stimulus” were common to *poly*Ala, *poly*Phe, *poly*Leu and hydrophobic LCR (Figs. S1A–S4A). The GO term “cellular process” was found in proteins with *poly*Ala, *poly*Leu, polar and hydrophobic LCRs (Figs. S2A–S5A). The processes “localization” and “locomotion” were specific to *poly*Ala, *poly*Leu and hydrophobic LCR (Figs. S2A–S4A); “developmental process” was specific to *poly*Ala and hydrophobic LCRs (Figs. S2A and S4A) whereas the “immune system process” and “biogenesis” to *poly*Leu and hydrophobic LCR (Figs. S3A and S4A). The processes “biological adhesion,” “biological regulation” and “reproduction” were exclusive for proteins with hydrophobic LCRs (Fig. S4A).

Molecular functions analysis in each category of LCRs showed involvement in “catalytic activity” of proteins with *poly*Ala, hydrophobic and polar LCRs (Figs. S2B, S4B and S5B). The MF term “receptor activity” was observed in proteins with *poly*Phe (Fig. S1B), *poly*Leu (Fig. S3B) and hydrophobic LCR (Fig. S4B) while “binding” was enriched in *poly*Ala, *poly*Leu and hydrophobic LCR. *poly*Phe and hydrophobic LCR were also associated with “signal transducer activity.” Two additional MF terms “structural molecule activity” and “transporter activity” were unique to hydrophobic LCR.

Furthermore, the CC analysis of amyloidogenic LCRs containing human protein suggested that *poly*Ala and hydrophobic LCR were common in “cell part,” “macromolecular complex” and “membrane” (Figs. S2C and S4C). The CC GO term “extracellular region” was found in proteins with *poly*Leu and hydrophobic LCR (Fig. S3C). In hydrophobic LCRs, we found additional components such as “extracellular matrix” and “organelle” (Fig. S4C).

We could not found GO slim BP for proteins containing *poly*Asn, *poly*Gln and *poly*Val which most likely occurred because GO slim provides a broad outline of GO contents. We then searched specific terms for the proteins in these categories from PANTHER. We found that proteins with amyloids in *poly*Gln had GO BP terms “ion transport” and “neutrophil degranulation” and for proteins with *poly*Val the terms were “G-protein coupled receptor signaling pathway,” “neuropeptide signaling pathway,” “female pregnancy” and “hormone metabolic process.” The MF term for *poly*Gln was “nucleic acid binding” and for *poly*Val these were “G-protein coupled receptor activity,” “neuropeptide Y receptor activity,” “protein binding” and “neuropeptide receptor activity.” We found that GO CC terms for *poly*Gln was “lysosomal membrane,” “microtubule organizing center,” “plasma membrane,” “integral component of membrane,” “specific granule membrane,” “intracellular membrane-bounded organelle,” “extracellular exosome” and “tertiary granule membrane.” Proteins containing amyloids in *poly*Val were associated to “plasma membrane” and “integral component of plasma membrane.”

PANTHER showed pathways for only *poly*Ala and hydrophobic LCRs. We found that proteins of both the categories were mainly associated to various signaling pathways (Table 2). The proteins forming amyloids in hydrophobic LCRs were also involved in many other pathways and cascades such as plasminogen activating cascade, cadherin signaling pathway, Wnt signaling pathway and Alzheimer disease-presenilin pathway (Table 2).

**Table 2:** Pathways and functional classes of human proteome with predicted aggregation tendency within low complexity regions.

Category	*poly*Ala	*poly*Leu	Polar	Hydrophobic
**Pathways**				
5HT2 type receptor mediated signaling pathway->SNARE Complex	–	–	–	NP_003752
5HT3 type receptor mediated signaling pathway->SNARE Complex	–	–	–	NP_003752
5HT4 type receptor mediated signaling pathway->SNARE Complex	–	–	–	NP_003752
Adrenaline and noradrenaline biosynthesis->amine translocator	–	–	–	NP_001003841
Alzheimer disease-presenilin pathway->Matrix metalloprotease	–	–	–	NP_004985, NP_071405
Angiogenesis->Phosphatidylinositol 3-kinase	NP_006210	–	–	–
Apoptosis signaling pathway->Phosphatidylinositol 3-kinase	NP_006210	–	–	–
Axon guidance mediated by netrin->Phosphatidylinositol 3-kinase	NP_006210	–	–	–
B cell activation->Phosphatidylinositol 3-kinase	NP_006210	–	–	–
Beta1 adrenergic receptor signaling pathway->SNARE Complex	–	–	–	NP_003752
Beta2 adrenergic receptor signaling pathway->SNARE Complex	–	–	–	NP_003752
Beta3 adrenergic receptor signaling pathway->SNARE Complex	–	–	–	NP_003752
Blood coagulation->Plasmin	–	–	–	NP_000292
Blood coagulation->Plasminogen	–	–	–	NP_000292
Cadherin signaling pathway->Cadherin	–	–	–	NP_061758
CCKR signaling map->MMP9	–	–	–	NP_004985
CCKR signaling map->p110	NP_006210	–	–	–
Cortocotropin releasing factor receptor signaling pathway->SNARE Complex	–	–	–	NP_003752
Dopamine receptor mediated signaling pathway->SNARE Complex	–	–	–	NP_003752
EGF receptor signaling pathway->Phosphatidylinositol 3-kinase	NP_006210	–	–	–
Endothelin signaling pathway->Adenylate cyclase	–	–	–	NP_001107
Endothelin signaling pathway->Phosphatidylinositol 3-kinase	NP_006210	–	–	–
FGF signaling pathway->fibroblast growth factor	–	–	–	NP_004456
FGF signaling pathway->Phosphatidylinositol 3-kinase	NP_006210	–	–	–
GABA-B receptor II signaling->adenylate cyclase	–	–	–	NP_001107
Heterotrimeric G-protein signaling pathway-Gi alpha and Gs alpha mediated pathway->Adenylyl cyclase	–	–	–	NP_001107
Heterotrimeric G-protein signaling pathway-Gi alpha and Gs alpha mediated pathway->Gs-protein coupled receptor	–	–	–	NP_001040, NP_001043
Heterotrimeric G-protein signaling pathway-Gi alpha and Gs alpha mediated pathway->Gi protein coupled receptor	–	–	–	NP_001040, NP_001043
Heterotrimeric G-protein signaling pathway-Gq alpha and Go alpha mediated pathway->Go-protein coupled receptor	–	–	–	NP_001040, NP_001043
Huntington disease->alpha-Adaptin	–	–	–	AAH14214
Hypoxia response via HIF activation->Phosphatidylinositol 3-kinase	NP_006210	–	–	–
Inflammation mediated by chemokine and cytokine signaling pathway->Chemokine receptor	–	–	–	NP_001286
Inflammation mediated by chemokine and cytokine signaling pathway->Phosphatidylinositol 3-kinase	NP_006210	–	–	–
Insulin/IGF pathway-protein kinase B signaling cascade->Phosphatidylinositol 3-kinase	NP_006210	–	–	–
Integrin signaling pathway->Phosphatidylinositol 3-kinase	NP_006210	–	–	–
Interleukin signaling pathway->Phosphatidylinositol 3-kinase	NP_006210	–	–	–
Ionotropic glutamate receptor pathway->N-ethylmaleimide-sensitive factor attachment protein receptor	–	–	–	NP_003752
Metabotropic glutamate receptor group II pathway->N-ethylmaleimide-sensitive factor attachment protein receptor	–	–	–	NP_003752
Metabotropic glutamate receptor group III pathway->N-ethylmaleimide-sensitive factor attachment protein receptor	–	–	–	NP_003752
Muscarinic acetylcholine receptor 1 and 3 signaling pathway->N-ethylmaleimide-sensitive factor attachment protein receptor	–	–	–	NP_003752
Muscarinic acetylcholine receptor 2 and 4 signaling pathway->N-ethylmaleimide-sensitive factor attachment protein receptor	–	–	–	NP_003752
Nicotinic acetylcholine receptor signaling pathway->N-ethylmaleimide-sensitive factor attachment protein receptor	–	–	–	NP_003752
Opioid proenkephalin pathway->SNARE Complex	–	–	–	NP_003752
Opioid proopiomelanocortin pathway->SNARE Complex	–	–	–	NP_003752
Opioid prodynorphin pathway->SNARE Complex	–	–	–	NP_003752
Oxytocin receptor mediated signaling pathway->SNARE Complex	–	–	–	NP_003752
p53 pathway->Phosphatidylinositol 3-kinase	NP_006210	–	–	–
PDGF signaling pathway->Phosphatidylinositol 3-kinase	NP_006210	–	–	–
PI3 kinase pathway->p110	NP_006210	–	–	–
PI3 kinase pathway->Activated p110	NP_006210	–	–	–
Plasminogen activating cascade->Plasmin	–	–	–	NP_000292
Plasminogen activating cascade->Plasminogen	–	–	–	NP_000292
Plasminogen activating cascade->pro-matrix metalloprotease 9	–	–	–	NP_004985
Ras Pathway->Phosphatidylinositol 3-kinase	NP_006210	–	–	–
T cell activation->Phosphatidylinositol 3-kinase	NP_006210	–	–	–
TGF-beta signaling pathway->Transforming growth factor beta	–	–	–	NP_004855
Thyrotropin-releasing hormone receptor signaling pathway->SNARE Complex	–	–	–	NP_003752
VEGF signaling pathway->Phosphatidylinositol 3-kinase	NP_006210	–	–	–
Wnt signaling pathway->Cadherin	–	–	–	NP_061758
Wnt signaling pathway->secreted frizzled-related protein	–	–	–	NP_003004
**Protein classes**				
Apolipoprotein (PC00052)	–	–	–	NP_085144
Aspartic protease (PC00053)	–	–	–	NP_116191
Cation transporter (PC00068)	–	–	–	NP_001003841, NP_001010893
Cell adhesion molecule (PC00069)	–	–	–	NP_005788, NP_658911, NP_057333
Chemokine (PC00074)	–	NP_001094282, NP_071342	–	NP_001094282, NP_071342
DNA binding protein (PC00009)	CAA64246, NP_004464, NP_663632	–	–	–
Enzyme modulator (PC00095)	–	–	–	NP_001010886
Glycosyltransferase (PC00111)	–	–	–	NP_065202
G-protein coupled receptor (PC00021)	–	–	–	NP_055694, NP_001074924, NP_001040, NP_000721, NP_003004, NP_001043
G-protein modulator (PC00022)	–	–	–	NP_853514
Growth factor (PC00112)	–	–	–	NP_004855, NP_004456
Homeodomain transcription factor (PC00119)	NP_663632	–	–	–
Ion channel (PC00133)	–	–	–	NP_853514
Immunoglobulin receptor superfamily (PC00124)	–	–	–	NP_002278, NP_068352, NP_055033, NP_065396, NP_055034
Intermediate filament binding protein (PC00130)	–	–	–	NP_001138241
Kinase (PC00137)	NP_006210	–	–	–
Membrane-bound signaling molecule (PC00152)	–	–	–	NP_002278, NP_068352, NP_853514, NP_055033, NP_065396, NP_055034
Metalloprotease (PC00153)	–	–	–	NP_003804, NP_004985, NP_071405
Non-receptor serine/threonine protein kinase (PC00167)	–	–	NP_079440	–
Protease (PC00190)	–	–	–	NP_853514
Protease inhibitor (PC00191)	–	–	–	NP_002278, NP_068352, NP_055033, NP_065396, NP_003004, NP_055034
Serine protease (PC00203)	–	–	–	NP_003782, NP_853514, NP_000292
Signaling molecule (PC00207)	–	–	–	NP_003004
Transmembrane receptor regulatory/adaptor protein (PC00226)	–	–	–	AAH14214
Transporter (PC00227)	–	–	–	NP_055557, NP_004791, NP_085144
Type I cytokine receptor (PC00231)	–	NP_851565, NP_851564, NP_068570	–	NP_851565, NP_851564, NP_068570
Voltage-gated sodium channel (PC00243)	–	–	–	NP_005788, NP_658911
Winged helix/forkhead transcription factor (PC00246)	CAA64246, NP_004464	–	–	–

We found protein class annotation for only the LCRs composed of *poly*Ala, *poly*Leu, polar and hydrophobic amino acids in human proteome. Proteins containing amyloids in *poly*Ala were kinase, DNA binding protein, and forkhead and homeodomain transcription factor (Table 2). Some of the proteins containing *poly*Leu were Type I cytokine receptor and Chemokine. Polar LCRs which formed amyloid in human proteins contained non-receptor serine/threonine kinase. In case of hydrophobic LCRs, proteins showed diversified class such as glycosyltransferase, chemokine, protease, receptor, enzyme modulator, signaling molecule and transporter (Table 2).

In *Data IIh* for validated amyloids in human proteins, we found only hydrophobic LCRs that were involved in amyloid formation. In PANTHER, MF of protein containing this LCR is “protein binding”; BP were “respiratory gaseous exchange” and “cellular protein metabolic process”; and CC were “extracellular region,” “extracellular space,” “endoplasmic reticulum membrane,” “integral component of membrane,” “lamellar body,” “clathrin-coated endocytic vesicle” and “multivesicular body lumen.”

## Discussion

This study outlines the correlation of LCR amino acid composition to their abundance, function and amyloidogenic properties. Herein, we separated LCRs into different sets: LCRs composed of (i) single amino acid repeats, (ii) positively charged amino acids, (iii) negatively charged amino acids, (iv) polar amino acids and (v) hydrophobic amino acids. We found that number of LCR subgroups varied widely across different subsets. Among LCRs containing repeats of single amino acids, most abundant was *poly*Gln followed by *poly*Asn (Table 1). Similar observation was reported earlier also (Faux et al., 2005). LCRs constituted of *poly*Cys, *poly*Met and aromatic acids were found to be very rare. Despite the fact Gln was the most abundant homopolymeric run, we found that total number of Asn residue was more. We feel this may be due to the reason that the *poly*Asn forms longer LCR stretches. When the abundance of LCR was analyzed on the basis of their physico-chemical properties, the highest number was observed of LCRs composed of polar amino acids followed by hydrophobic, negatively charged and positively charged LCRs.

Further, we analyzed the aggregation propensity in different LCR subgroups. On prediction of amyloids in LCRs it was observed that whereas *poly*Ala, *poly*Ile, *poly*Leu, *poly*Phe, *poly*Asn, *poly*Gln, polar and hydrophobic LCRs had high potential to exhibit amyloidogenic nature, *poly*Asp, *poly*Glu, *poly*Gly, *poly*Lys and charged LCRs were least amyloidogenic. All of these are the major LCR groups. We also observed that complete stretch of *poly*Ile and *poly*Phe were predicted to be involved in amyloid formation. In addition, when we analyzed the nature of experimentally validated amyloids, we found that validated amyloids were also rich in Gln, Asn, Ala and Ser.

Biological functions were found to be highly diverse in LCR-containing proteins such as signal transduction, RNA biosynthetic process, protein localization, cellular response to stress, chromosome organization, cell division, peptidoglycan metabolic process, and transport. In addition, we also noticed involvement of proteins with LCRs in transcription, metal ion binding and nucleic acid binding. Since these functions are also observed in disordered proteins, hence it suggests the association of amyloidogenic LCRs in disordered proteins.

We also functionally analyzed the annotation of the human proteins that showed amyloid formation in LCRs. We found that amyloids were predicted in only *poly*Ala, *poly*Phe, *poly*Leu, *poly*Asn, *poly*Gln, *poly*Val, hydrophobic and polar LCRs of human proteins. Our analysis showed that human proteins containing these amyloid forming LCRs were mostly involved in biological regulation and cellular processes. The major MF of human proteins predicted with amyloidogenic LCRs was binding. Whereas the LCR-containing proteins were related to lumen and plasma membrane but human proteins in which LCR was predicted as amyloidogenic, were present only in membrane. Some of the BP were absent in homopolymeric runs but appeared when they were forming LCRs in combination with other amino acid(s). For example, the process “reproduction” was seen in hydrophobic LCR-containing proteins but this process was absent in *poly*Leu (hydrophobic amino acids).

## Conclusions

We have grouped LCRs on the basis of physico-chemical properties of amino acid and analyzed their composition, functions and amyloidogenic behavior. We found that Gly runs were mostly involved in translation while proteins having other LCRs were involved in transcription. Our analysis on 40% redundant SwissProt proteins indicated that LCRs composed of polar and hydrophobic amino acids are the most common and predicted to form amyloids. But analysis on human proteome showed that most of the amyloidogenic LCRs were composed of hydrophobic amino acids. Also, the combination of amino acids of same physico-chemical properties in LCRs results in gain-of-function in the corresponding protein. We hope establishment of correlation between LCR and amyloid propensity in a protein will help in our understanding of diseases caused by protein misfolding and aggregation.

## Supplemental Information

10.7717/peerj.5823/supp-1 Supplemental Information 1 (a) Biological processes and (b) molecular functions of *poly*Phe in human proteins predicted to form amyloids.

10.7717/peerj.5823/supp-2 Supplemental Information 2 (a) Biological processes (b) molecular functions and (c) cellular components of *poly*Ala in human proteins predicted to form amyloids.

10.7717/peerj.5823/supp-3 Supplemental Information 3 (a) Biological processes (b) molecular functions and (c) cellular components of *poly*Leu in human proteins predicted to form amyloids.

10.7717/peerj.5823/supp-4 Supplemental Information 4 (a) Biological processes (b) molecular functions and (c) cellular components of LCRs in human proteins containing combination of hydrophobic amino acids predicted to form amyloids.

10.7717/peerj.5823/supp-5 Supplemental Information 5 (a) Biological processes and (b) molecular functions of LCRs in human proteins containing combination of polar amino acids predicted to form amyloids.

10.7717/peerj.5823/supp-6 Supplemental Information 6 Top 5 molecular functions of proteins containing perfect single amino acid repeats and combination of amino acids having similar physicochemical group.

10.7717/peerj.5823/supp-7 Supplemental Information 7 Top 5 biological processes of proteins containing perfect single amino acid repeats and combination of amino acids having similar physicochemical group.

10.7717/peerj.5823/supp-8 Supplemental Information 8 Top 5 cellular components of proteins containing perfect single amino acid repeats and combination of amino acids having similar physicochemical group.

## Additional Information and Declarations

### Data Availability

The following information was supplied regarding data availability: The raw data is available in the Supplemental Files.
