# Supplementary figures and images for: Comparative functional analysis of proteins containing low-complexity predicted amyloid regions
Source: PeerJ. 2018 Oct 30;6:e5823. doi: 10.7717/peerj.5823 (PMC6214233; doi:10.7717/peerj.5823)

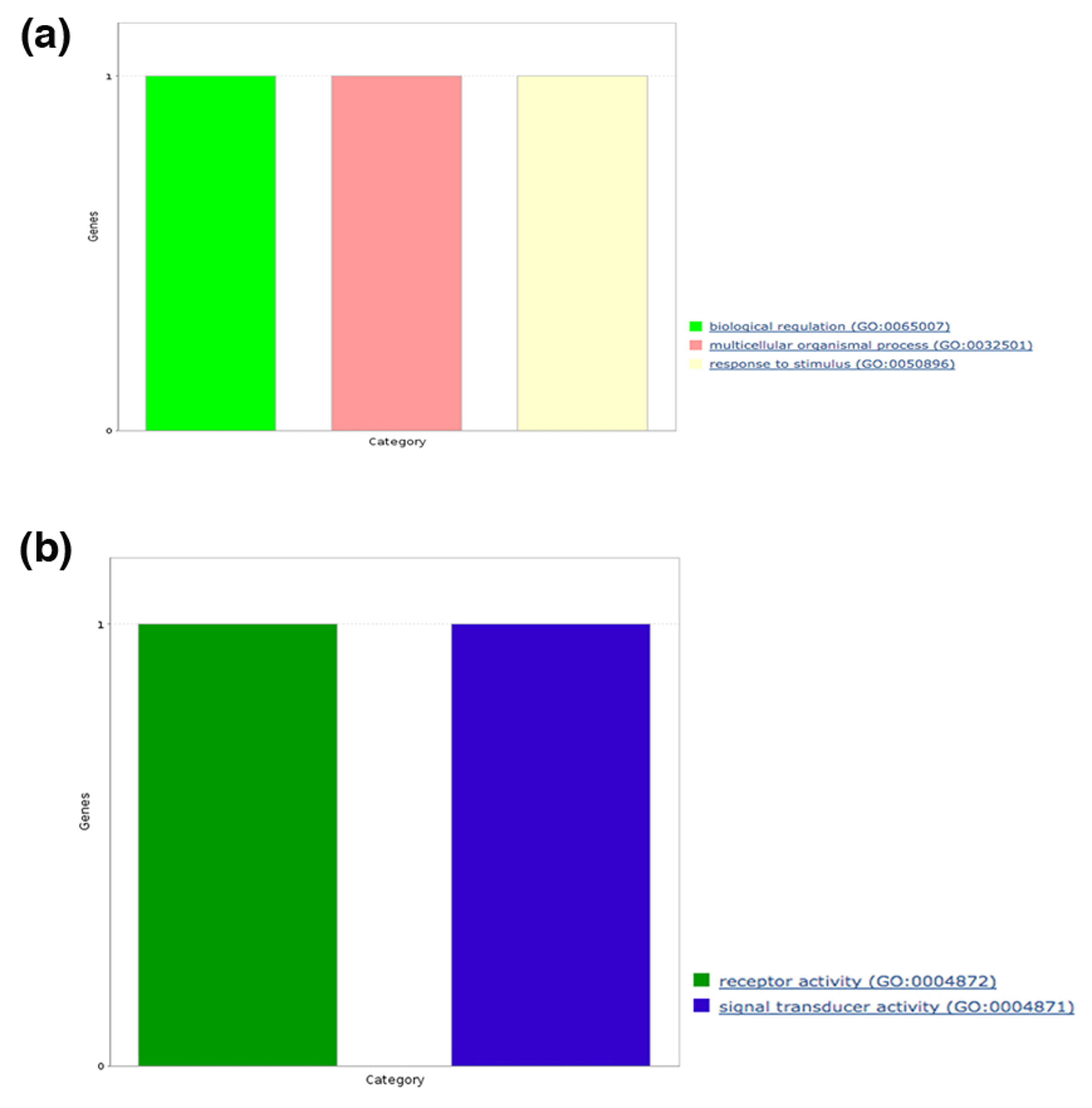

Supplement: Supplemental Information 1 [file peerj-06-5823-s001.png]

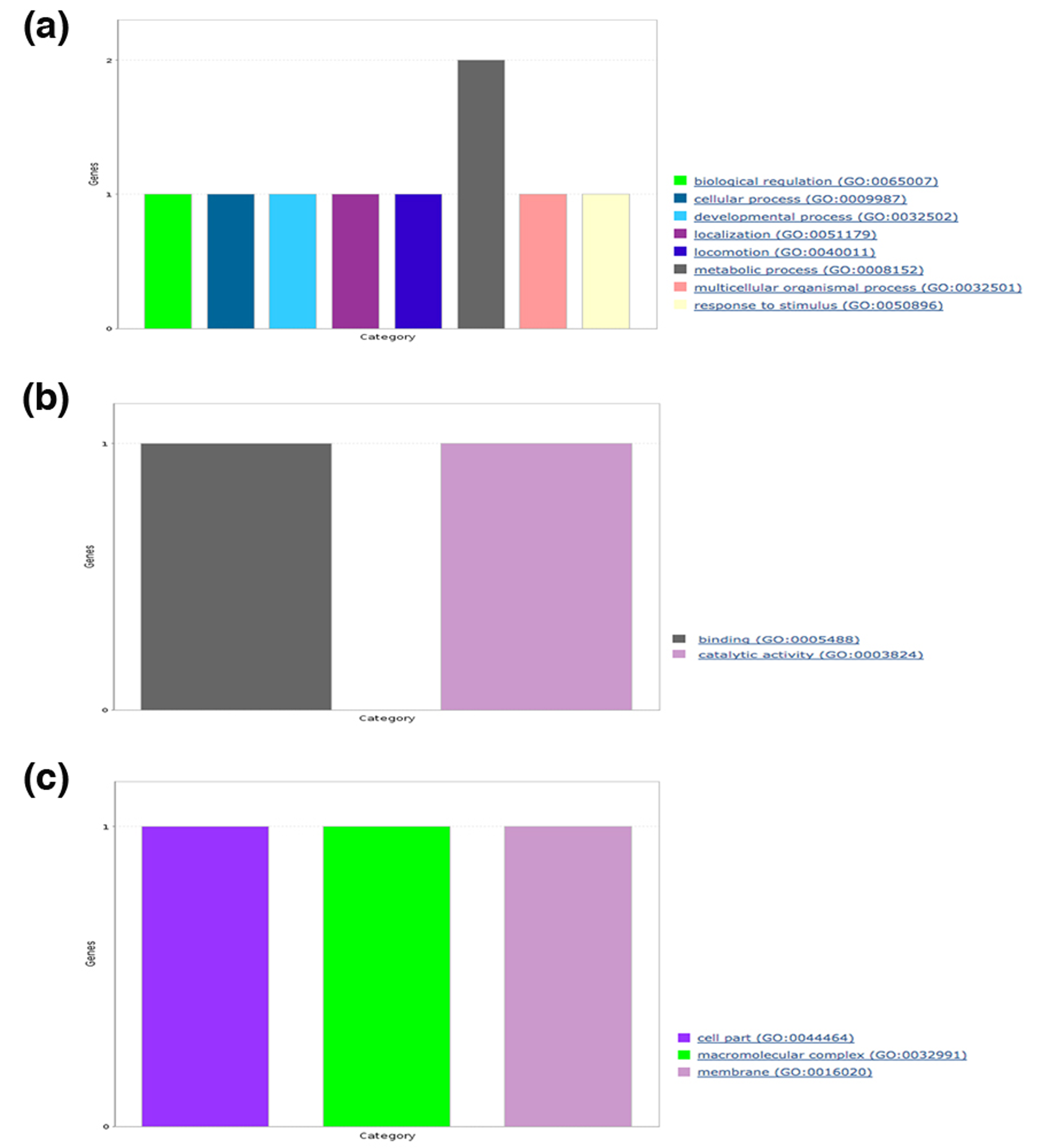

Supplement: Supplemental Information 2 [file peerj-06-5823-s002.png]

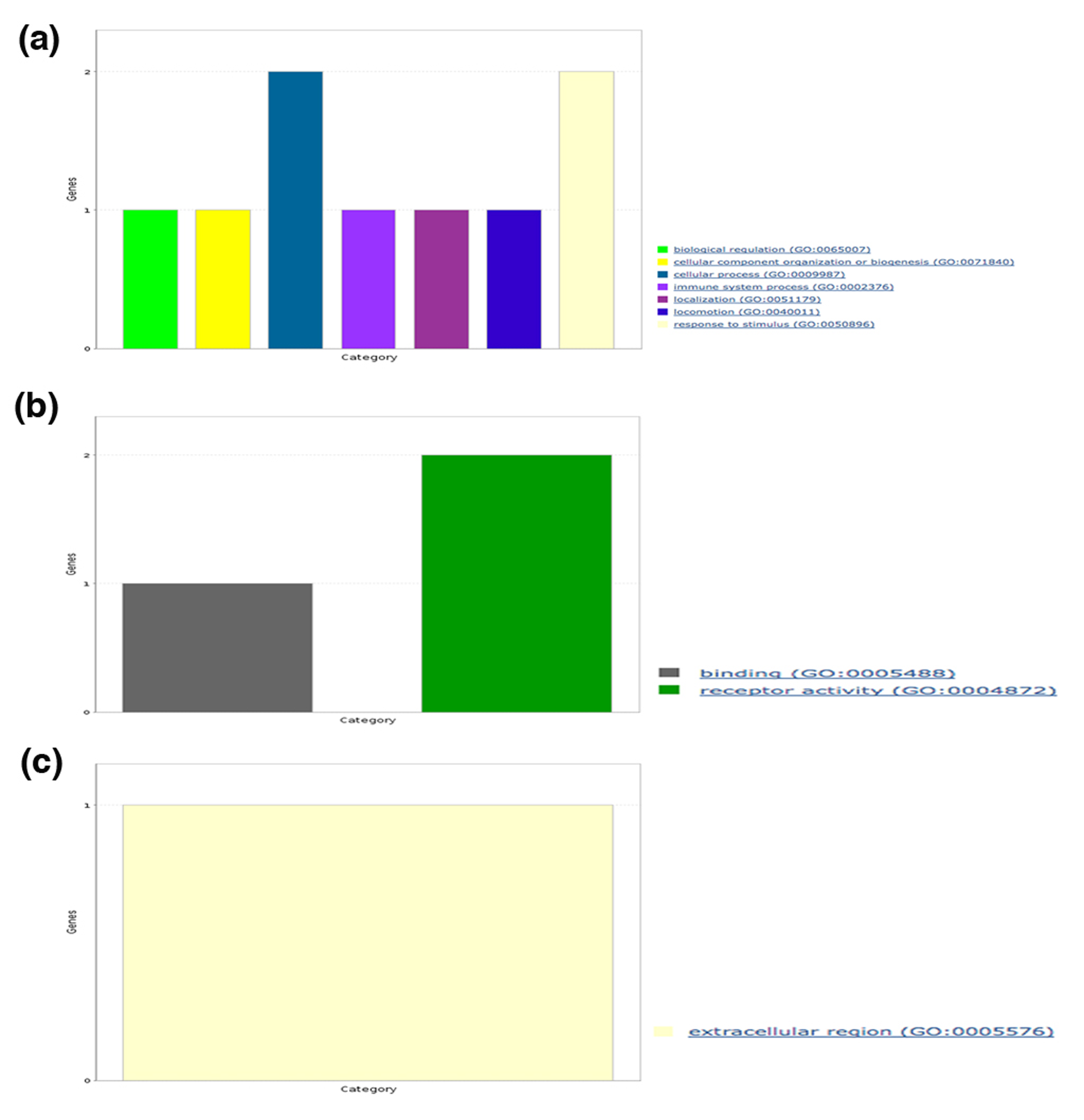

Supplement: Supplemental Information 3 [file peerj-06-5823-s003.png]

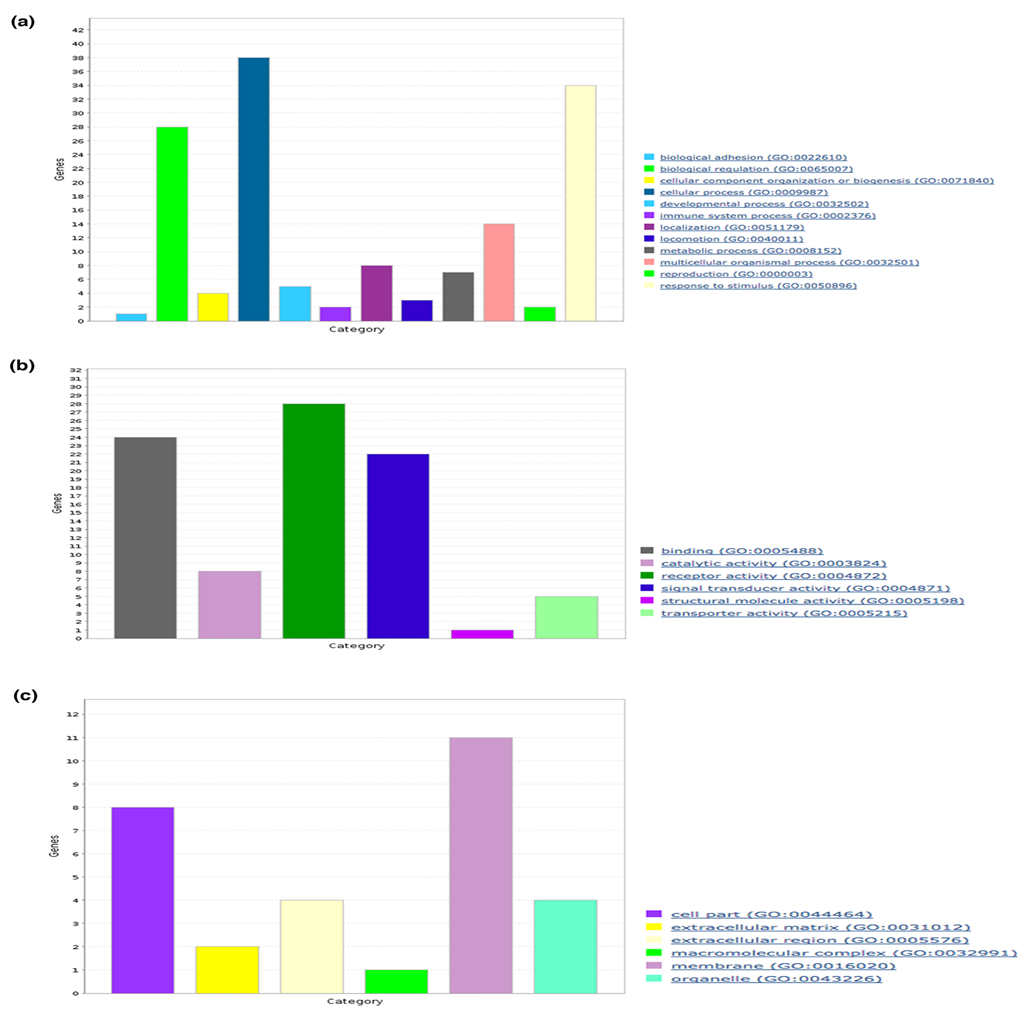

Supplement: Supplemental Information 4 [file peerj-06-5823-s004.png]

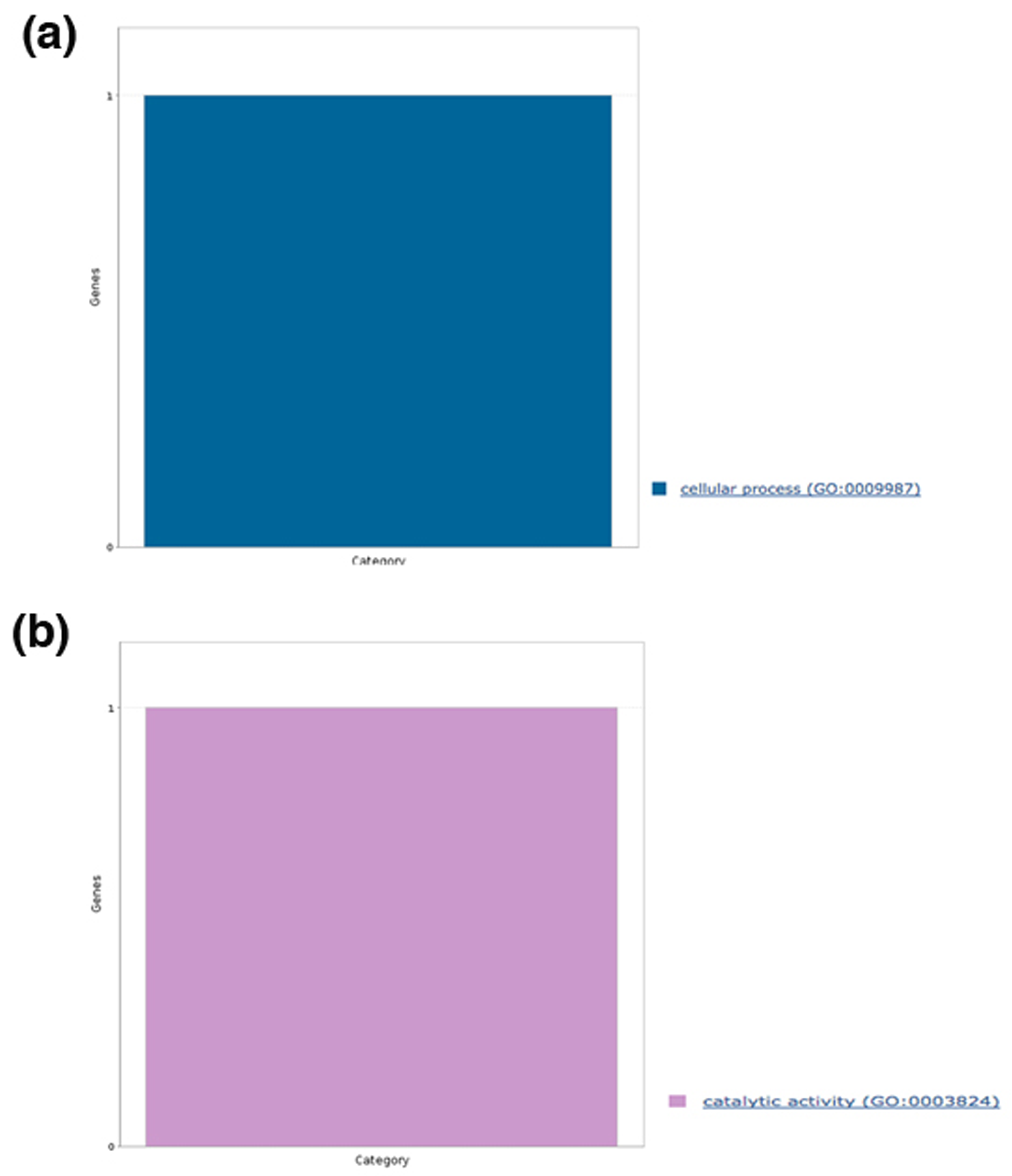

Supplement: Supplemental Information 5 [file peerj-06-5823-s005.png]
