# Supplementary material for: Comparative functional analysis of proteins containing low-complexity predicted amyloid regions
Source: PeerJ. 2018 Oct 30;6:e5823. doi: 10.7717/peerj.5823 (PMC6214233; doi:10.7717/peerj.5823)
Supplement: Supplemental Information 6 [file peerj-06-5823-s006.docx]

**Table S1: Top 5 molecular functions of proteins containing perfect single amino acid repeats and combination of amino acids having similar physicochemical group.**

| **GO terms** | **Description** | **Count** | **P-value** | **Benjamini** |
| --- | --- | --- | --- | --- |
| ***Alanine repeats*** | | | | |
| GO:0043169 | Cation binding | 149 | 8.9E-6 | 4.3E-4 |
| GO:0043167 | Ion binding | 149 | 1.2E-5 | 5.1E-4 |
| GO:0046872 | Metal ion binding | 148 | 4.9E-6 | 2.6E-4 |
| GO:0003677 | DNA binding | 131 | 4.2E-26 | 1.0E-23 |
| GO:0046914 | Transition metal ion binding | 113 | 7.8E-7 | 4.7E-5 |
| ***Glycine repeats*** | | | | |
| GO:0017076 | Purine nucleotide binding | 71 | 5.1E-2 | 4.0E-1 |
| GO:0030554 | Adenyl nucleotide binding | 69 | 7.4E-3 | 9.6E-2 |
| GO:0001883 | Purine nucleoside binding | 69 | 7.6E-3 | 9.4E-2 |
| GO:0001882 | Nucleoside binding | 69 | 9.7E-3 | 1.1E-1 |
| ***Proline repeats*** | | | | |
| GO:0043167 | Ion binding | 73 | 1.4E-4 | 3.1E-3 |
| GO:0043169 | Cation binding | 72 | 2.2E-4 | 3.8E-3 |
| GO:0046872 | Metal ion binding | 71 | 2.1E-4 | 4.1E-3 |
| GO:0046914 | Transition metal ion binding | 62 | 3.4E-7 | 1.1E-5 |
| GO:0008270 | Zinc ion binding | 61 | 8.7E-18 | 5.8E-16 |
| ***Serine repeats*** | | | | |
| GO:0003677 | DNA binding | 69 | 1.8E-9 | 1.6E-7 |
| GO:0046914 | Transition metal ion binding | 65 | 6.4E-3 | 1.3E-1 |
| ***Threonine repeats*** | | | | |
| GO:0000166 | Nucleotide binding | 48 | 5.5E-3 | 6.5E-2 |
| GO:0017076 | Purine nucleotide binding | 44 | 6.4E-3 | 7.2E-2 |
| GO:0030554 | Adenyl nucleotide binding | 43 | 9.8E-4 | 1.7E-2 |
| GO:0001883 | Purine nucleoside binding | 43 | 1.0E-3 | 1.6E-2 |
| GO:0001882 | Nucleoside binding | 43 | 1.3E-3 | 1.9E-2 |
| ***Histidine repeats*** | | | | |
| GO:0003677 | DNA binding | 31 | 1.7E-16 | 5.1E-15 |
| GO:0030528 | Transcription regulator activity | 27 | 1.7E-20 | 1.1E-18 |
| GO:0003700 | Transcription factor activity | 23 | 6.8E-20 | 2.4E-18 |
| GO:0043565 | Sequence-specific DNA binding | 16 | 2.7E-14 | 4.7E-13 |
| GO:0046914 | Transition metal ion binding | 15 | 3.2E-2 | 2.2E-1 |
| ***Aspartic Acid repeats*** | | | | |
| GO:0046872 | Metal ion binding | 30 | 8.7E-2 | 7.0E-1 |
| ***Glutamic Acid repeats*** | | | | |
| GO:0046872 | Metal ion binding | 98 | 1.8E-6 | 1.1E-4 |
| GO:0043169 | Cation binding | 98 | 4.3E-6 | 2.0E-4 |
| GO:0043167 | Ion binding | 98 | 5.3E-6 | 2.2E-4 |
| GO:0003677 | DNA binding | 69 | 2.2E-11 | 8.4E-9 |
| GO:0008270 | Zinc ion binding | 60 | 3.5E-11 | 6.6E-9 |
| ***Asparagine repeats*** | | | | |
| GO:0003677 | DNA binding | 31 | 1.6E-9 | 8.0E-8 |
| GO:0030528 | Transcription regulator activity | 29 | 3.3E-16 | 5.1E-14 |
| ***Glutamine repeats*** | | | | |
| GO:0003677 | DNA binding | 86 | 2.1E-23 | 2.2E-21 |
| GO:0030528 | Transcription regulator activity | 77 | 7.9E-39 | 2.4E-36 |
| ***Cysteine repeats*** | | | | |
| **-** | **-** | **-** | **-** | **-** |
| ***Lysine repeats*** | | | | |
| **-** | - | - | - | - |
| ***Arginine repeats*** | | | | |
| GO:0043169 | Cation binding | 31 | 3.4E-3 | 6.6E-2 |
| GO:0043167 | Ion binding | 31 | 3.6E-3 | 6.3E-2 |
| GO:0046872 | Metal ion binding | 30 | 5.2E-3 | 8.0E-2 |
| GO:0003677 | DNA binding | 28 | 2.7E-8 | 1.5E-6 |
| GO:0046914 | Transition metal ion binding | 25 | 9.7E-4 | 2.2E-2 |
| ***Leucine repeats*** | | | | |
| ***-*** | - | - | - | - |
| ***Isoleucine repeats*** | | | | |
| ***-*** | - | - | - | - |
| ***Methionine repeats*** | | | | |
| ***-*** | - | - | - | - |
| ***Valine repeats*** | | | | |
| ***-*** | - | - | - | - |
| ***Phenylalanine repeats*** | | | | |
| ***-*** | - | - | - | - |
| ***Tryptophan repeats*** | | | | |
| ***-*** | - | - | - | - |
| ***Tyrosine repeats*** | | | | |
| ***-*** | - | - | - | - |
|  |  |  |  |  |
| **LCRs containing combination of positively charged amino acids** | | | | |
| GO:0003677 | DNA binding | 17.4 | 5.4E-4 | 4.6E-2 |
| GO:0005515 | Protein binding | 15.7 | 2.3E-2 | 4.4E-1 |
| GO:0003700 | Transcription factor activity, sequence-specific DNA binding | 8.4 | 9.9E-3 | 2.9E-1 |
| GO:0044822 | poly(A) RNA binding | 6.2 | 1.4E-4 | 2.3E-2 |
| GO:0004674 | Protein serine/threonine kinase activity | 5.6 | 1.1E-2 | 2.8E-1 |
| **LCRs containing combination of negatively charged amino acids** | | | | |
| GO:0005515 | Protein binding | 17.1 | 2.9E-6 | 1.2E-3 |
| GO:0046872 | Metal ion binding | 12.9 | 3.4E-2 | 4.6E-1 |
| GO:0003677 | DNA binding | 12.5 | 1.3E-2 | 3.0E-1 |
| GO:0008270 | Zinc ion binding | 7.6 | 2.2E-2 | 3.5E-1 |
| GO:0000166 | Nucleotide binding | 7.1 | 2.4E-3 | 1.3E-1 |
| **LCRs containing combination of polar amino acids** | | | | |
| GO:0003677 | DNA binding | 13.8 | 1.2E-12 | 1.8E-10 |
| GO:0005524 | ATP binding | 13.8 | 1.1E-6 | 9.8E-5 |
| GO:0005515 | Protein binding | 12.8 | 3.4E-4 | 1.6E-2 |
| GO:0046872 | Metal ion binding | 11.8 | 5.2E-3 | 1.2E-1 |
| GO:0000166 | Nucleotide binding | 10.5 | 1.9E-38 | 1.7E-35 |
| **LCRs containing combination of hydrophobic amino acids** | | | | |
| GO:0016787 | Hydrolase activity | 4.3 | 1.2E–2 | 2.8E–1 |
| GO:0005509 | Calcium ion binding | 3.8 | 2.6E–5 | 5.5E–3 |
| GO:0004872 | Receptor activity | 2.3 | 3.4E–7 | 1.5E–4 |
| GO:0004252 | Serine-type endopeptidase activity | 2.3 | 2.7E–5 | 2.9E–3 |
| GO:0004930 | G-protein coupled receptor activity | 2.1 | 1.1E–3 | 5.2E–2 |
