# Supplementary material for: Comparative functional analysis of proteins containing low-complexity predicted amyloid regions
Source: PeerJ. 2018 Oct 30;6:e5823. doi: 10.7717/peerj.5823 (PMC6214233; doi:10.7717/peerj.5823)
Supplement: Supplemental Information 7 [file peerj-06-5823-s007.docx]

**Table S2: Top 5 biological processes of proteins containing perfect single amino acid repeats and combination of amino acids having similar physicochemical group.**

| **GO terms** | **Description** | **Count** | **P-value** | **Benjamini** |
| --- | --- | --- | --- | --- |
| ***Alanine repeats*** | | | | |
| GO:0045449 | Regulation of Transcription | 132 | 1.0E-41 | 1.6E-38 |
| GO:0006350 | Transcription | 111 | 2.6E-30 | 2.0E-27 |
| GO:0051252 | Regulation of RNA metabolic process | 81 | 3.5E-23 | 1.8E-20 |
| GO:0006355 | Regulation of Transcription, DNA-dependent | 79 | 1.7E-22 | 6.5E-20 |
| ***Glycine repeats*** | | | | |
| GO:0006412 | Translation | 52 | 2.2E-4 | 1.6E-2 |
| GO:0034660 | ncRNA metabolic process | 45 | 1.3E-7 | 1.7E-5 |
| GO:0006418 | tRNA aminoacylation for protein translation | 44 | 2.3E-19 | 1.0E-16 |
| GO:0043038 | Amino acid activation | 44 | 2.3E-19 | 7.2E-17 |
| GO:0043039 | tRNA aminoacylation | 44 | 2.3E-19 | 7.2E-17 |
| ***Proline repeats*** | | | | |
| GO:0045449 | Regulation of Transcription | 62 | 9.8E-22 | 1.1E-18 |
| GO:0006350 | Transcription | 46 | 4.2E-12 | 1.2E-9 |
| GO:0006355 | Regulation of Transcription, DNA-dependent | 39 | 4.0E-13 | 2.2E-10 |
| GO:0051252 | Regulation of RNA metabolic process | 39 | 7.6E-13 | 2.8E-10 |
| GO:0007010 | Cytoskeleton organization | 17 | 1.7E-10 | 2.7E-8 |
| ***Serine repeats*** | | | | |
| GO:0045449 | Regulation of Transcription | 72 | 2.7E-17 | 4.0E-14 |
| GO:0006350 | Transcription | 62 | 4.9E-13 | 1.8E-10 |
| GO:0051252 | Regulation of RNA metabolic process | 42 | 1.2E-8 | 2.9E-6 |
| GO:0006355 | Regulation of Transcription, DNA-dependent | 39 | 1.7E-7 | 2.7E-5 |
| GO:0006796 | Phosphate metabolic process | 31 | 5.0E-3 | 8.0E-2 |
| ***Threonine repeats*** | | | | |
| GO:0007049 | Cell cycle | 36 | 4.7E-18 | 1.1E-16 |
| GO:0051301 | Cell division | 34 | 4.0E-20 | 1.2E-18 |
| GO:0000270 | Peptidoglycan metabolic process | 33 | 5.5E-26 | 2.8E-23 |
| GO:0030203 | Glycosaminoglycan metabolic process | 33 | 1.3E-25 | 3.3E-23 |
| GO:0022604 | Regulation of cell morphogenesis | 33 | 1.9E-25 | 1.6E-23 |
| ***Histidine repeats*** | | | | |
| GO:0045449 | Regulation of Transcription | 31 | 5.4E-20 | 2.6E-17 |
| GO:0006350 | Transcription | 25 | 5.6E-14 | 1.4E-11 |
| GO:0006355 | Regulation of Transcription, DNA-dependent | 21 | 3.3E-13 | 5.3E-11 |
| GO:0051252 | Regulation of RNA metabolic process | 21 | 4.9E-13 | 5.9E-11 |
| GO:0006357 | Regulation of Transcription from RNA polymerase II promoter | 9 | 3.0E-7 | 2.1E-5 |
| ***Aspartic Acid repeats*** | | | | |
| GO:0006350 | Transcription | 19 | 3.1E-4 | 7.9E-2 |
| GO:0045449 | Regulation of Transcription | 17 | 4.4E-3 | 1.2E-1 |
| GO:0006396 | RNA processing | 13 | 2.8E-2 | 3.9E-1 |
| GO:0006259 | DNA metabolic process | 13 | 2.3E-2 | 3.6E-1 |
| GO:0008104 | Protein localization | 12 | 3.3E-3 | 1.1E-1 |
| ***Glutamic Acid repeats*** | | | | |
| GO:0045449 | Regulation of Transcription | 78 | 1.7E-23 | 2.1E-20 |
| GO:0006355 | Regulation of Transcription, DNA-dependent | 35 | 1.1E-6 | 2.2E-4 |
| GO:0051252 | Regulation of RNA metabolic process | 35 | 1.8E-6 | 3.0E-4 |
| GO:0007049 | Cell cycle | 30 | 6.0E-5 | 2.5E-3 |
| GO:0033554 | Cellular response to stress | 27 | 1.7E-3 | 3.3E-2 |
| ***Asparagine repeats*** | | | | |
| GO:0045449 | Regulation of Transcription | 37 | 3.6E-19 | 2.1E-16 |
| GO:0006350 | Transcription | 30 | 2.0E-13 | 5.6E-11 |
| GO:0006355 | Regulation of Transcription, DNA-dependent | 25 | 6.1E-13 | 1.2E-10 |
| GO:0051252 | Regulation of RNA metabolic process | 25 | 9.7E-13 | 1.4E-10 |
| ***Glutamine repeats*** | | | | |
| GO:0045449 | Regulation of Transcription | 107 | 2.3E-52 | 3.2E-49 |
| GO:0006350 | Transcription | 85 | 9.4E-35 | 3.2E-32 |
| GO:0051252 | Regulation of RNA metabolic process | 77 | 6.4E-39 | 4.4E-36 |
| GO:0006355 | Regulation of Transcription, DNA-dependent | 75 | 1.1E-37 | 5.2E-35 |
| GO:0051276 | Chromosome organization | 26 | 7.2E-13 | 7.5E-11 |
| ***Cysteine repeats*** | | | | |
| **-** | **-** | **-** | **-** | **-** |
| ***Lysine repeats*** | | | | |
| GO:0006350 | Transcription | 29 | 3.2E-9 | 1.6E-6 |
| GO:0045449 | Regulation of Transcription | 18 | 4.5E-3 | 2.4E-1 |
| GO:0006351 | Transcription, DNA-dependent | 12 | 1.4E-6 | 3.4E-4 |
| GO:0032774 | RNA biosynthetic process | 12 | 2.5E-6 | 4.1E-4 |
| GO:0006355 | Regulation of Transcription, DNA-dependent | 12 | 1.9E-2 | 4.9E-1 |
| ***Arginine repeats*** | | | | |
| GO:0045449 | Regulation of Transcription | 30 | 3.0E-13 | 1.8E-10 |
| GO:0006355 | Regulation of Transcription, DNA-dependent | 14 | 1.1E-4 | 9.7E-3 |
| GO:0051252 | Regulation of RNA metabolic process | 14 | 1.4E-4 | 1.0E-2 |
| GO:0007166 | Cell surface receptor linked signal transduction | 11 | 3.7E-5 | 5.6E-3 |
| ***Leucine repeats*** | | | | |
| ***-*** | - | - | - | - |
| ***Isoleucine repeats*** | | | | |
| ***-*** | - | - | - | - |
| ***Methionine repeats*** | | | | |
| ***-*** | - | - | - | - |
| ***Valine repeats*** | | | | |
| GO:0008610 | Lipid biosynthetic process | 4 | 9.5E-2 | 9.6E-1 |
| GO:0008654 | Phospholipid biosynthetic process | 3 | 5.5E-2 | 1.0E0 |
| GO:0006644 | Phospholipid metabolic process | 3 | 7.8E-2 | 9.9E-1 |
| GO:0019637 | Organophosphate metabolic process | 3 | 9.5E-2 | 9.8E-1 |
| ***Phenylalanine repeats*** | | | | |
| ***-*** | - | - | - | - |
| ***Tryptophan repeats*** | | | | |
| ***-*** | - | - | - | - |
| ***Tyrosine repeats*** | | | | |
| ***-*** | - | - | - | - |
|  |  |  |  |  |
| **LCRs containing combination of positively charged amino acids** | | | | |
| GO:0006355 | Regulation of transcription, DNA-templated | 21.9 | 1.9E–8 | 9.1E–6 |
| GO:0006351 | Transcription, DNA-templated | 20.8 | 3.2E–5 | 7.5E–3 |
| GO:0007275 | Multicellular organism development | 6.2 | 1.2E–2 | 6.9E–1 |
| GO:0006468 | Protein phosphorylation | 5.6 | 1.4E–2 | 6.0E–1 |
| GO:0045944 | Positive regulation of transcription from RNA polymerase II promoter | 5.1 | 5.9E–2 | 9.3E–1 |
| **LCRs containing combination of negatively charged amino acids** | | | | |
| GO:0006351 | Transcription, DNA-templated | 16.6 | 1.2E-5 | 7.8E-3 |
| GO:0006355 | Regulation of transcription, DNA-templated | 14.3 | 4.5E-6 | 6.0E-3 |
| GO:0015031 | Protein transport | 4.2 | 4.5E-2 | 8.0E-1 |
| GO:0000122 | Negative regulation of transcription from RNA polymerase II promoter | 3.7 | 1.5E-3 | 3.9E-1 |
| GO:0006281 | DNA repair | 3.4 | 6.8E-2 | 8.3E-1 |
| **LCRs containing combination of polar amino acids** | | | | |
| GO:0006351 | Transcription, DNA-templated | 16.0 | 8.3E-19 | 6.2E-16 |
| GO:0006355 | Regulation of transcription, DNA-templated | 14.0 | 1.0E-21 | 1.0E-18 |
| GO:0006468 | Protein phosphorylation | 5.6 | 1.4E-22 | 2.1E-19 |
| GO:0016310 | Phosphorylation | 4.9 | 2.3E-28 | 6.9E-25 |
| GO:0006810 | Transport | 4.4 | 5.5E-2 | 8.2E-1 |
| **LCRs containing combination of hydrophobic amino acids** | | | | |
| GO:0006810 | Transport | 5.4 | 1.2E–2 | 4.9E–1 |
| GO:0007165 | Signal transduction | 3.4 | 4.1E–2 | 7.5E–1 |
| GO:0006508 | Proteolysis | 3.0 | 1.2E–3 | 1.7E–1 |
| GO:0055085 | Transmembrane transport | 3.0 | 6.3E–3 | 3.7E–1 |
| GO:0045087 | Innate immune response | 2.7 | 2.1E–5 | 1.4E–2 |
