# Supplementary material for: Comparative functional analysis of proteins containing low-complexity predicted amyloid regions
Source: PeerJ. 2018 Oct 30;6:e5823. doi: 10.7717/peerj.5823 (PMC6214233; doi:10.7717/peerj.5823)
Supplement: Supplemental Information 8 [file peerj-06-5823-s008.docx]

**Table S3: Top 5 cellular components of proteins containing perfect single amino acid repeats and combination of amino acids having similar physicochemical group.**

| **GO terms** | **Description** | **Count** | **P-value** | **Benjamini** |
| --- | --- | --- | --- | --- |
| ***Alanine repeats*** | | | | |
| GO:0070013 | Intracellular organelle lumen | 31 | 1.0E-5 | 8.4E-4 |
| GO:0043233 | Organelle lumen | 31 | 1.2E-5 | 7.5E-4 |
| GO:0031974 | Membrane-enclosed lumen | 31 | 1.9E-5 | 9.0E-4 |
| ***Glycine repeats*** | | | | |
| GO:0070013 | Intracellular organelle lumen | 20 | 1.0E-3 | 9.7E-2 |
| GO:0043233 | Organelle lumen | 20 | 1.2E-3 | 7.3E-2 |
| GO:0031974 | Membrane-enclosed lumen | 20 | 1.5E-3 | 7.0E-2 |
| GO:0031981 | Nuclear lumen | 19 | 1.6E-4 | 3.0E-2 |
| GO:0005654 | Nucleoplasm | 11 | 3.6E-3 | 9.5E-2 |
| ***Proline repeats*** | | | | |
| GO:0043232 | Intracellular non-membrane-bounded organelle | 41 | 1.8E-2 | 1.1E-1 |
| GO:0043228 | Non-membrane-bounded organelle | 41 | 1.8E-2 | 1.1E-1 |
| GO:0044459 | Plasma membrane part | 36 | 2.8E-13 | 3.2E-11 |
| GO:0005856 | Cytoskeleton | 31 | 1.0E-14 | 2.3E-12 |
| ***Serine repeats*** | | | | |
| GO:0031974 | Membrane-enclosed lumen | 23 | 4.0E-2 | 5.1E-1 |
| GO:0031090 | Organelle membrane | 21 | 8.5E-2 | 6.1E-1 |
| ***Threonine repeats*** | | | | |
| GO:0009536 | Plastid | 6 | 7.2E-2 | 7.3E-1 |
| ***Histidine repeats*** | | | | |
| GO:0009536 | Plastid | 6 | 7.2E-2 | 7.3E-1 |
| GO:0070013 | Intracellular organelle lumen | 6 | 2.7E-3 | 4.9E-2 |
| GO:0043233 | Organelle lumen | 6 | 2.8E-3 | 3.4E-2 |
| GO:0031974 | Membrane-enclosed lumen | 6 | 3.1E-3 | 2.8E-2 |
| ***Aspartic Acid repeats*** | | | | |
| GO:0070013 | Intracellular organelle lumen | 16 | 3.4E-5 | 2.3E-3 |
| GO:0043233 | Organelle lumen | 16 | 3.8E-5 | 1.7E-3 |
| GO:0031974 | Membrane-enclosed lumen | 16 | 4.9E-5 | 1.6E-3 |
| ***Glutamic Acid repeats*** | | | | |
| GO:0070013 | Intracellular organelle lumen | 46 | 1.1E-13 | 3.0E-11 |
| GO:0043233 | Organelle lumen | 46 | 1.5E-13 | 2.0E-11 |
| GO:0031974 | Membrane-enclosed lumen | 46 | 3.1E-13 | 2.9E-11 |
| GO:0031981 | Nuclear lumen | 39 | 1.5E-12 | 1.0E-10 |
| GO:0005654 | Nucleoplasm | 23 | 2.7E-8 | 1.5E-6 |
| ***Asparagine repeats*** | | | | |
| GO:0005694 | Chromosomal part | 5 | 1.1E-2 | 2.9E-1 |
| ***Glutamine repeats*** | | | | |
| GO:0031974 | Membrane-enclosed lumen | 41 | 3.1E-15 | 1.9E-13 |
| GO:0031981 | Nuclear lumen | 39 | 1.3E-17 | 1.0E-15 |
| GO:0070013 | Intracellular organelle lumen | 39 | 3.5E-14 | 1.7E-12 |
| GO:0043233 | Organelle lumen | 39 | 4.6E-14 | 1.9E-12 |
| ***Cysteine repeats*** | | | | |
| **-** | **-** | **-** | **-** | **-** |
| ***Lysine repeats*** | | | | |
| GO:0043232 | Intracellular non-membrane-bounded organelle | 42 | 1.8E-5 | 5.2E-4 |
| GO:0043228 | Non-membrane-bounded organelle | 42 | 1.8E-5 | 4.5E-4 |
| GO:0031981 | Nuclear lumen | 32 | 2.3E-16 | 1.9E-14 |
| GO:0070013 | Intracellular organelle lumen | 32 | 1.8E-13 | 1.1E-11 |
| GO:0043233 | Organelle lumen | 32 | 2.3E-13 | 1.0E-11 |
| ***Arginine repeats*** | | | | |
| **-** | **-** | **-** | **-** | **-** |
| ***Leucine repeats*** | | | | |
| GO:0044459 | Plasma membrane part | 32 | 4.8E-4 | 1.4E-2 |
| GO:0031226 | Intrinsic to plasma membrane | 17 | 1.2E-4 | 5.6E-3 |
| GO:0000267 | Cell fraction | 15 | 6.9E-4 | 1.6E-2 |
| ***Isoleucine repeats*** | | | | |
| ***-*** | - | - | - | - |
| ***Methionine repeats*** | | | | |
| ***-*** | - | - | - | - |
| ***Valine repeats*** | | | | |
| GO:0016021 | Integral to membrane | 14 | 1.7E-2 | 2.5E-1 |
| GO:0031224 | Intrinsic to membrane | 14 | 2.5E-2 | 2.5E-1 |
| GO:0005886 | Plasma membrane | 12 | 1.7E-2 | 4.4E-1 |
| ***Phenylalanine repeats*** | | | | |
| ***-*** | - | - | - | - |
| ***Tryptophan repeats*** | | | | |
| ***-*** | - | - | - | - |
| ***Tyrosine repeats*** | | | | |
| ***-*** | - | - | - | - |
|  |  |  |  |  |
| **LCRs containing combination of positively charged amino acids** | | | | |
| GO:0005634 | Nucleus | 44.9 | 1.3E-8 | 2.5E-6 |
| GO:0005730 | Nucleolus | 12.9 | 2.0E-8 | 1.9E-6 |
| GO:0030054 | Cell junction | 3.9 | 9.1E-3 | 4.4E-1 |
| GO:0005694 | Chromosome | 2.8 | 8.4E-2 | 8.1E-1 |
| GO:0005623 | Cell | 2.2 | 4.4E-2 | 8.2E-1 |
| **LCRs containing combination of negatively charged amino acids** | | | | |
| GO:0005634 | Nucleus | 42.0 | 4.1E-20 | 1.6E-17 |
| GO:0005737 | Cytoplasm | 27.3 | 1.8E-4 | 1.6E-2 |
| GO:0005730 | Nucleolus | 8.6 | 7.7E-11 | 1.4E-8 |
| GO:0005654 | Nucleoplasm | 6.5 | 4.5E-4 | 3.3E-2 |
| GO:0005622 | Intracellular | 4.2 | 8.9E-2 | 7.5E-1 |
| **LCRs containing combination of polar amino acids** | | | | |
| GO:0005634 | Nucleus | 36.8 | 1.1E-50 | 8.5E-48 |
| GO:0005737 | Cytoplasm | 23.6 | 1.8E-6 | 4.6E-4 |
| GO:0016020 | Membrane | 12.6 | 3.1E-4 | 2.5E-2 |
| GO:0005730 | Nucleolus | 5.7 | 3.1E-14 | 1.2E-11 |
| GO:0005654 | Nucleoplasm | 5.1 | 5.9E-6 | 7.3E-4 |
| **LCRs containing combination of hydrophobic amino acids** | | | | |
| GO:0016021 | Integral component of membrane | 60.1 | 6.2E–66 | 1.9E–63 |
| GO:0005886 | Plasma membrane | 30.2 | 2.0E–16 | 3.3E–14 |
| GO:0016020 | Membrane | 20.2 | 1.7E–9 | 1.3E–7 |
| GO:0005576 | Extracellular region | 11.4 | 1.0E–7 | 6.1E–6 |
| GO:0005887 | Integral component of plasma membrane | 9.8 | 1.7E–12 | 1.7E–10 |
